# Generators of Phenotypic Diversity in the Evolution of Pathogenic Microorganisms

**DOI:** 10.1371/journal.ppat.1003181

**Published:** 2013-03-21

**Authors:** Silvia Calo, R. Blake Billmyre, Joseph Heitman

**Affiliations:** Department of Molecular Genetics and Microbiology, Duke University Medical Center, Durham, North Carolina, United States of America; Washington University School of Medicine, United States of America

## Introduction

**Figure 1:**
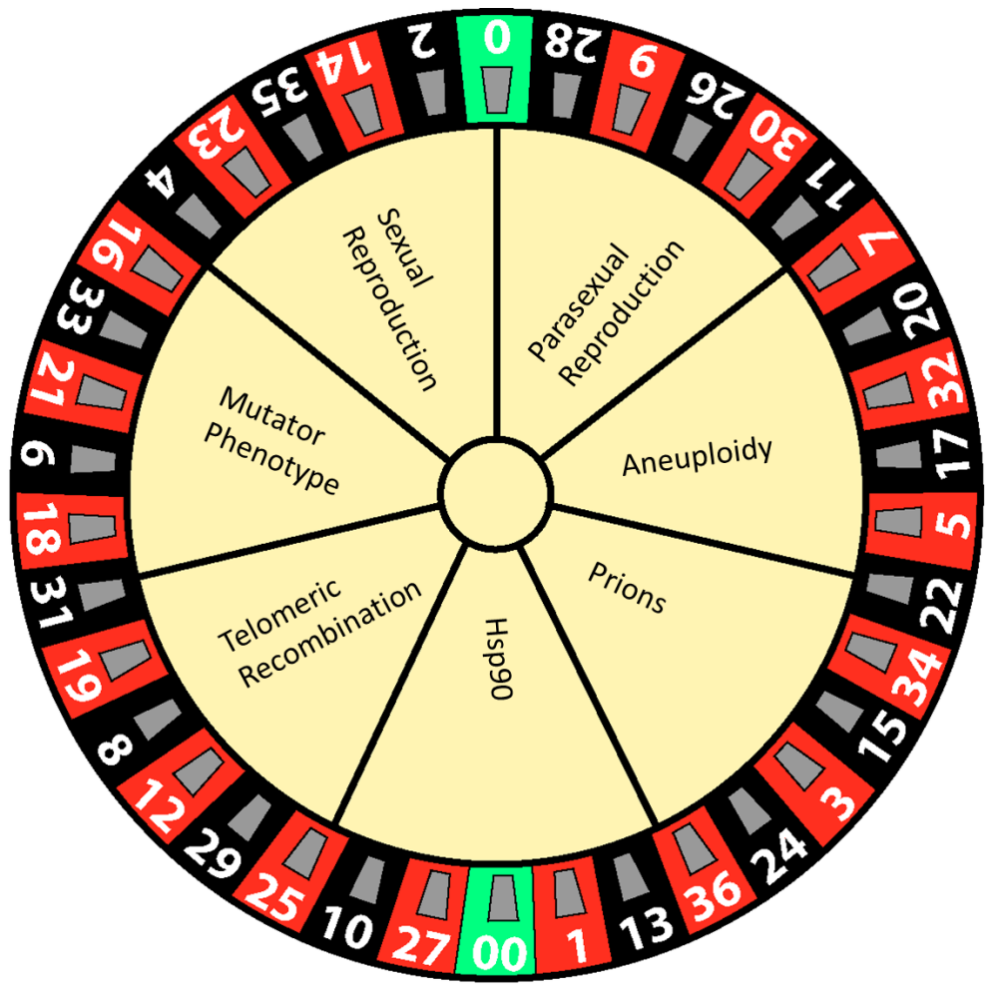
Generators of phenotypic diversity illustrated as a game of chance. Evolution is a multifactorial process that depends on numerous complex factors, including routes to diversity that can be deleterious, neutral, or advantageous depending on the environment. Here we depict these evolutionary trajectories using the analogy of a roulette wheel in which one possible path yields a winner that develops a new trait that allows the microorganism to survive in novel conditions, such as a host treated with antimicrobial agents. However, as in roulette, the odds are against the gambler and the other routes and outcomes are deleterious.

All organisms run the gauntlet of Darwinian selection. Poignant examples include microbial pathogens, which must survive and thrive in their hosts. The process of pathogen adaptation to the host is diverse and is now known to involve a panoply of diversity generators, such as sexual/parasexual reproduction, aneuploidy, prions, mutators, telomeric silencing/recombination, and Hsp90 as a capacitor for evolution [1], [2] (Figure 1). Given the vast diversity of known mechanisms that can generate phenotypic and genotypic assortments, others likely remain to be discovered.

## Sexual and Parasexual Reproduction Create Diversity

Most eukaryotic microorganisms are either known or suspected to undergo sexual reproduction. However, as recently as two decades ago, most pathogenic eukaryotic microbes, including fungi and parasites, were thought to be clonal and asexual [3], [4]. Over the past decade, we have witnessed a renaissance in this field and now appreciate that most, and perhaps all, pathogenic fungi and parasites have retained sexual capacity [5], [6]. In many cases, their sexual cycles can be difficult to observe, due to being cryptic or having a unisexual or even parasexual cycle. These modes of reproduction share the ability to promote some level of genetic exchange, but involve inbreeding or even selfing in many instances, helping to preserve well-adapted genomic configurations while simultaneously generating limited genetic diversity that may promote adaptation to less rapidly changing host or environmental niches.

Pathogenic microbes can reproduce parasexually or sexually. Parasexuality involves cell–cell fusion and ploidy reduction through stochastic, random chromosome loss. This phenomenon was originally described by Pontecorvo for *Aspergillus nidulans* [7], and has been more recently described in *Candida albicans* by Forche and colleagues [8]. Parasex can produce genetic diversity via independent chromosomal assortment, mitotic recombination, and the ability of the diploid state to act as a capacitor for evolution by enabling the accumulation of recessive mutations that are deleterious individually but beneficial in combination (so-called reciprocal sign epistasis) [9].

Sexual reproduction can accelerate evolution by purging the genome of deleterious mutations or by bringing together combinations of advantageous alleles. Opposite-sex mating promotes genetic exchange via outcrossing, whereas unisexual reproduction can involve inbreeding or selfing to yield more limited genetic exchange. The capacity to engage in opposite sexual, unisexual, and asexual reproduction may be a bet-hedging strategy that enables microbes to better adapt to a range of environments, including the host. The fact that two of the most common human fungal pathogens, *C. albicans* and *Cryptococcus neoformans*, have retained the capacity for a sexual cycle involving opposite-sex mating as well as the capability of unisexual reproduction, provides an illustration of convergent evolution [10], [11]. Recent discoveries on the sexual nature of parasitic pathogens, including examples of unisexuality and selfing [12], [13], unify this paradigm across the two major groups of eukaryotic pathogens.

## Aneuploidy: Deleterious or Advantageous?

Aneuploidy can be deleterious, which is exemplified in common human genetic diseases, such as Down's syndrome, and in cancers. However, aneuploidy also can be advantageous. In fungi, aneuploidy confers antifungal drug resistance and enables rapid adaptive evolution. In addition, these findings may extend to protozoan parasites.

In *C. albicans*, the most common human fungal pathogen, treating patients with fluconazole results in the rapid emergence of drug-resistant aneuploid isolates [14]. These azole-resistant isolates harbor a novel isochromosome containing two left arms of chromosome 5. Notably, this amplified genomic region includes two key genes: *ERG11*, which encodes lanosterol 14 alpha demethylase, the target of azole antifungal drugs, and *TAC1*, which encodes a transcription factor that activates drug efflux pump expression. Strikingly, a similar type of aneuploidy underlies azole resistance in *C. neoformans*, the second most common human systemic fungal pathogen [15]. In this example, disomy of chromosome 1 is responsible for azole heteroresistance and remarkably contains two key target genes: one encodes Erg11 and the second encodes Afr1, a major efflux pump for azoles. Recent studies in *Saccharomyces cerevisiae* further underscore a central role for aneuploidy in enabling rapid adaptive evolution and also reveal novel phenotypes associated with aneuploidy [16], [17]. Additionally, mutations have been identified that allow strains to better tolerate aneuploidy by enabling the turnover of otherwise deleterious proteins in stoichiometric imbalance [18].

The impact of aneuploidy extends beyond model and pathogenic fungi to parasitic pathogens. Recent studies reveal that populations of the protozoan parasite *Leishmania* are ensembles of different ploidy states, including individuals that are monosomic, disomic, or trisomic for different chromosomes [19], [20]. The resulting state has been termed mosaic aneuploidy [21] and may contribute to drug resistance and promote pathogenesis, analogous to fungal azole resistance, by enabling genotypic and thereby phenotypic diversification.

## Hsp90 as a Capacitor for Evolution

The Hsp90 chaperone system alters relationships between genotypes and phenotypes under conditions of environmental stress, and thereby plays a role in evolutionary processes and provides a route to genetically complex traits in a single mechanistic step [22].

Populations contain silent genetic variation, which can be buffered by chaperones such as the heat-shock protein Hsp90. Hsp90 interacts with, and maintains in their active state, a diverse set of “client” proteins, many of which are signal-transducing kinases or transcription factors involved in cell cycle and developmental regulation. Minor changes in amino acid sequence could have important effects on conformational stability or function of these regulatory proteins as well as a wide range of other proteins. Hsp90 recognizes characteristic structures rather than specific sequences, and is therefore able to chaperone these unstable proteins. In this way, Hsp90 buffers genotypic variation, allowing diversity to accumulate in a latent form under neutral conditions. General protein damage, or moderate changes in growth conditions such as heat stress, diverts Hsp90 from its usual targets to different, partially denatured proteins, uncovering morphological variants that are then expressed under these conditions. Eventually, these variants can become fixed genetic traits independent of chaperone regulation or loss. This surprising role for Hsp90 as a capacitor for morphological evolution and phenotypic variation is conserved across the fungal, plant, and animal kingdoms [1], [2], [23]. Hsp90 can also act as a potentiator of variability by: 1) chaperoning mutated cell regulators that are prone to misfolding, or 2) through its interaction with the cell signaling regulator calcineurin, allowing new traits such as drug resistance to appear in a diverse range of fungal species [23].

## Prions Can Drive Evolution

Prions were originally discovered via their ability to cause disease in mammals, including spongiform encephalopathies such as Kuru and fatal familial insomnia, and were found to be unusual, infectious, or inheritable variant forms of a host protein. Prions are also known to occur in fungal species where they can also be deleterious [24], [25]. However, prions can provide mechanisms to unveil preexisting variation. One such protein that can become a prion, Sup35, is an *S. cerevisiae* translation termination factor. Like other prion-forming proteins, Sup35 contains an N-terminal domain that is dispensable for the normal function of the protein and can occasionally adopt an amyloid conformation, converting the protein to its prion state [*PSI^+^*]. When this occurs, Sup35 forms inactive complexes sequestering most of the protein and increasing the frequency of stop codon readthrough to result in proteins with novel C-terminal extensions. The ability to switch to the [*PSI^+^*] state can thereby provide a temporary survival advantage under diverse conditions by exposing previously concealed genetic variation. This was observed when cells from different genetic backgrounds were grown as the [*PSI^+^*] or [*psi^−^*] state in more than 150 phenotypic assays, including inhibitors of diverse cellular processes, general stress conditions, and different temperatures [26], [27]. The advantageous switch to the [*PSI^+^*] state expands the population size of organisms with this phenotypic state, increasing the likelihood that this new trait may be fixed in the population as a result of subsequent genetic change [28]. By connecting protein homeostasis with stress responses, prions can drive phenotypic plasticity and thereby allow cells to grow under new conditions without necessarily preventing them from surviving in their former environment [29].

## Recombinant Telomeres Cloak Microbial Pathogens

Telomeric and subtelomeric regions are locations whose genomic content can be rapidly restructured. Trypanosomes (unicellular, parasitic, and flagellated protozoa) capitalize on this capacity as part of their pathogenic strategy. The trypanosome cell surface is highly immunogenic, but rapid switching to different surface glycoproteins enables immune evasion. The trypanosome genome encodes more than 1,000 different surface glycoproteins, but only one is expressed at any given time by virtue of its location at one of approximately 15 different subtelomeric expression sites. Two of the three predominant mechanisms for switching result from recombination between telomeres, either through homologous recombination or gene conversion [30]. Similarly, in the pathogenic fungus *Candida glabrata*, a large family of *EPA* adhesin genes are clustered in subtelomeric genomic regions, and most of these genes are silenced by a *SIR3*-dependent telomeric silencing pathway. While the loss of any individual member of the *EPA* family confers little phenotypic change, loss of an entire cluster decreases pathogenicity, suggesting redundancy between different family members [31].

In other pathogens, such the plant pathogenic fungus *Magnaporthe grisea*, virulence determinants are also clustered in subtelomeric regions. Approximately 50% of identified *M. grisea* avirulence genes are located in subtelomeres [32]. Avirulence genes typically mediate host invasion, but are individually recognized by hosts with the correct immune receptors. Loss or inactivation of an avirulence gene can therefore increase pathogenicity under some circumstances, while retention is favored in other situations. The telomeres of rye grass–infecting strains of *M. grisea* are unstable during growth, which may serve as a mechanism to silence or delete avirulence genes [33].

## Mutator States Diversify Genomic Repertoire

Diversity can also be generated through the development of a “mutator” state in which the frequency of mutations is dramatically increased. Inactivation of mismatch repair or loss of oxidative stress protection enzymes can create heritable mutator phenotypes by destabilizing the genome. Alternatively, transient mutator states are also possible. Two examples include the induction of the SOS response in *Escherichia coli* and the stochastic mistranslation of DNA polymerase in a single cell, resulting in errors in DNA replication [34], [35]. While the mutations created by a transient mutator are heritable, the hypermutability state itself is not.

Typically hypermutability would be seen as evolutionarily disfavored, but in the short term, it can enable adaptation to a new, previously inhospitable environment. For example, the lungs of cystic fibrosis patients are often infected with *Pseudomonas aeruginosa*. Progression of cystic fibrosis injures the lungs, which results in a constantly changing environment for the pathogen. A substantial proportion of chronic *P. aeruginosa* infections are caused by mutator strains, while acute infections are not, suggesting that mutability potentiates long-term adaptation [36]. In fungi, loss of mismatch repair genes like *MSH2* can result in destabilization of repeat tracts [37]. This enables alteration of adhesin genes in *S. cerevisiae*, many of which contain repeat tracts [38]. Intragenic repeats are also present in the cell surface genes of a number of other pathogens, including *Aspergillus fumigatus*, *C. albicans*, and *Plasmodium* [39]. The majority of hypermutator studies have been conducted in bacteria, and further study in pathogenic fungi and parasites is an area ripe for exploration, as illustrated by a recent study implicating mutator action in *C. neoformans* colony/cell morphology variation selected by growth with amoeba [40].

Many of the pathways that cause advantageous mutator phenotypes in bacteria or yeasts are implicated in oncogenesis in mammals. Destabilization of repeat tracts in yeast can be adaptive, but loss of the *MSH2* homolog in humans results in hereditary nonpolyposis colon cancer [41], [42]. Likewise, transient mutators are thought to be responsible for some of the multiple hit mutations required for carcinogenesis [43].

Microorganisms are able to evolve via all of the resources they have available, even when many routes seem detrimental at first glance. Here we have endeavored to summarize recent advances to provide a broad vision of this topic. Other examples have not been covered because of space limitations, such as genetic noise or transposon movement, and probably even more will be discovered in the future, since we know from Lewis Carroll that “it takes all the running you can do, to keep in the same place.”
